# Machine Learning for Autism Spectrum Disorder Prediction: A Review of Data Augmentation and Feature Selection Techniques

**DOI:** 10.1002/hcs2.70098

**Published:** 2026-09-07

**Authors:** Sahar Alkhaibari, Feng Dong

**Affiliations:** ^1^ Department of Computer and Information Sciences University of Strathclyde Glasgow United Kingdom; ^2^ Department of Computer Science University of Tabuk Tabuk Saudi Arabia

**Keywords:** autism spectrum disorder, data augmentation, deep learning, early diagnosis, feature selection, machine learning, neurodevelopmental disorders

## Abstract

Autism spectrum disorder (ASD) is a complex neurodevelopmental condition characterized by persistent difficulties in social communication, social interaction, and repetitive behaviors. Early and accurate diagnosis is essential but is often hindered by subjective clinical assessments, limited data availability, and inconsistencies in existing diagnostic tools. This review evaluates the role of machine learning and deep learning approaches in improving ASD prediction, with a particular focus on two important yet relatively underexplored methodological components: data augmentation and feature selection. A structured literature search was conducted across major scientific databases, including IEEE Xplore, PubMed, Scopus, and Google Scholar, to identify studies published between 2021 and 2024. The methodological quality and risk of bias of the included studies were assessed using the Prediction Model Risk of Bias Assessment Tool. A total of 26 peer‐reviewed studies were included based on their relevance to machine learning/deep learning‐based ASD prediction and their explicit application of data augmentation or feature selection techniques. Data augmentation methods were categorized into conventional approaches, such as geometric and color‐space transformations, and advanced techniques, including generative adversarial network‐based synthetic data generation. Although augmentation techniques may improve model robustness and help address dataset scarcity, relatively few studies conducted ablation analyses to isolate the contribution of individual augmentation strategies. Feature selection approaches were classified into filter, wrapper, and embedded methods. Commonly used techniques included information gain, chi‐square tests, recursive feature elimination, and elastic net regularization. While these methods may improve predictive performance and model interpretability, they are frequently applied without sufficient empirical justification or biological interpretation. Overall, this review highlights important methodological limitations, including limited external validation, insufficient ablation analyses, and inadequate evaluation frameworks, which reduce confidence in reported performance improvements and model generalizability. Future research should emphasize methodological transparency, robust validation strategies, multimodal data integration, and clinically interpretable modeling approaches to improve the reliability and clinical applicability of ASD prediction systems.

## Graphical abstract

Autism spectrum disorder (ASD) is a complex neurodevelopmental condition characterized by persistent difficulties in social communication, social interaction, and repetitive behaviors. Early diagnosis is often hindered by subjective clinical assessments and limited data availability. This review evaluates machine learning and deep learning approaches in improving ASD prediction, focusing on data augmentation and feature selection. A structured literature search was conducted across IEEE Xplore, PubMed, Scopus, and Google Scholar for studies published between 2021 and 2024. Twenty‐six peer‐reviewed studies were included. Data augmentation methods were categorized into conventional approaches (geometric and color‐space transformations) and advanced techniques (generative adversarial network‐based synthetic data generation). Feature selection approaches were classified into filter, wrapper, and embedded methods. This review highlights important methodological limitations, including limited external validation, insufficient ablation analyses, and inadequate evaluation frameworks, which reduce confidence in reported performance and model generalizability. Future research should emphasize methodological transparency, robust validation, multimodal data integration, and clinically interpretable modeling.

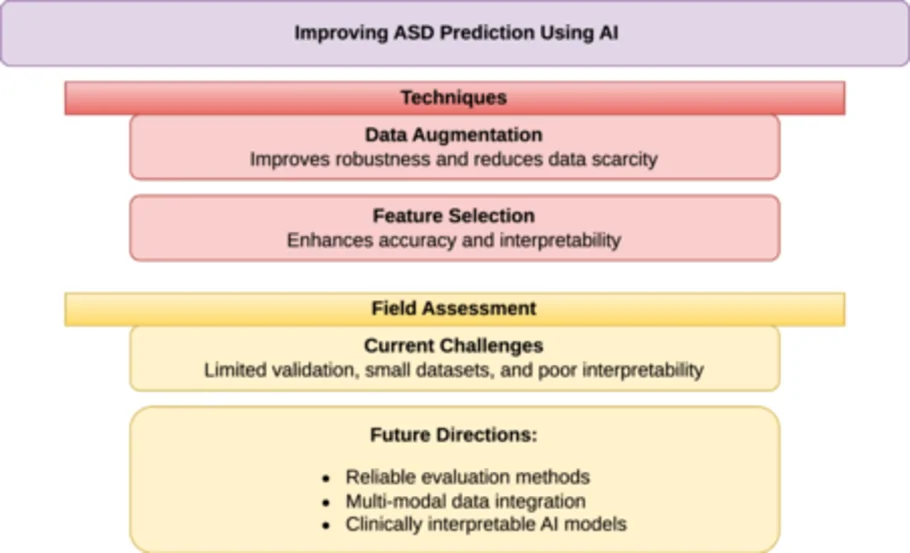

**Abbreviations**
AI: artificial intelligence
ASD: autism spectrum disorder
AUC: area under the receiver operating characteristic curve
CNN: convolutional neural network
DL: deep learning
EEG: electroencephalography
GAN: generative adversarial network
LSTM: long short‐term memory
ML: machine learning
MRI: magnetic resonance imaging
PCA: principal component analysis
PROBAST: Prediction Model Risk of Bias Assessment Tool
RF: random forest
RFE: recursive feature elimination
RFECV: recursive feature elimination with cross‐validation
RNN: recurrent neural network
ROB: risk of bias
rs‐fMRI: resting‐state functional magnetic resonance imaging
SFFS: sequential forward feature selection
SVM: support vector machine

## 1 Background

Autism spectrum disorder (ASD) is a neurodevelopmental condition characterized by difficulties in social communication, restricted interests, and repetitive behaviors [1, 2]. Although reliable diagnosis is possible by the age of 2 years [2], many children are not diagnosed until between 4 and 5 years of age [3], delaying access to early intervention and support services.

ASD affects a substantial proportion of the global child population [4]. Early screening and diagnosis are important for improving developmental outcomes [4]; however, conventional diagnostic approaches face several limitations [5]. The absence of definitive biomarkers [6], restricted access to specialized healthcare services [7], and reliance on subjective clinical assessments contribute to diagnostic delays and inconsistencies [2]. Widely used diagnostic tools, including the Autism Diagnostic Interview, Autism Diagnostic Observation Schedule, Screening Tool for Autism in Toddlers and Young Children, and Childhood Autism Rating Scale‐2, are frequently criticized for being time‐consuming, costly, and highly dependent on expert evaluation [8, 9]. In some regions, the waiting period for a formal ASD diagnosis may exceed 3 years [8].

In response to the limitations of traditional diagnostic methods, machine learning (ML) and deep learning (DL) techniques are increasingly being explored for their potential to improve the early detection of ASD. As subfields of artificial intelligence (AI), ML and DL have been widely adopted in healthcare applications, including disease detection, medical image analysis, and personalized treatment planning [10]. In ASD research, these approaches can automate aspects of diagnosis, reduce associated costs, and support clinical decision‐making through the analysis of complex medical and behavioral data [8, 11]. ML methods have shown considerable promise in tasks such as risk assessment, classification, and feature extraction, particularly when applied to modalities including magnetic resonance imaging (MRI), electroencephalography (EEG), eye‐tracking, and facial imaging data [12]. Despite these advantages, important challenges remain, including concerns related to data privacy, algorithmic bias, and regulatory compliance [13].

Two important techniques that enhance the performance and reliability of ML models in ASD prediction are data augmentation and feature selection. These approaches play a central role in improving model accuracy, interpretability, and generalizability, particularly in the presence of limited sample sizes, class imbalance, and high‐dimensional datasets commonly encountered in ASD research.

Data augmentation refers to the synthetic expansion of training datasets through transformations or generative approaches. In image‐based ASD studies, augmentation techniques may include geometric transformations such as rotation, flipping, scaling, and color‐space modifications applied to facial or neuroimaging data [14, 15]. These techniques are especially important in ASD research because available datasets are often small, imbalanced, and heterogeneous. Small datasets are particularly susceptible to overfitting, where models memorize training examples rather than learning generalizable patterns, ultimately reducing predictive performance on unseen data [16, 17]. Data augmentation helps mitigate these limitations by increasing dataset diversity and encouraging models to learn more robust decision boundaries. This is particularly important in DL applications for medical imaging, where annotated datasets are often scarce, expensive to obtain, and variable across acquisition protocols and image resolutions [15, 16].

Feature selection involves identifying and retaining the most informative input variables while removing irrelevant, redundant, or noisy features [18]. This process reduces dataset dimensionality, improves computational efficiency, enhances model interpretability, and helps mitigate the “curse of dimensionality,” whereby excessive feature numbers relative to sample size may reduce predictive performance. In ASD prediction studies, input data may include clinical, behavioral, neuroimaging, and genetic variables. Consequently, feature selection plays an important role in identifying informative biomarkers, such as specific EEG patterns, facial characteristics, or questionnaire responses, that contribute meaningfully to ASD classification. In addition, feature selection may improve the interpretability of predictive models for clinicians and researchers by highlighting the variables most strongly associated with ASD.

This review examines recent ML and DL studies in ASD prediction, with a particular focus on the influence of data augmentation and feature selection techniques on model performance. Specifically, this review addresses the following research questions:

(1) What data augmentation and feature selection techniques are commonly used in ASD‐focused ML/DL models?

(2) How do these techniques influence model accuracy, generalizability, and overfitting in ASD prediction tasks?

Understanding the methodological strategies used in the existing literature is essential for identifying best practices and guiding the development of future predictive models. Although numerous reviews have examined ML applications in ASD diagnosis, most have focused primarily on comparing algorithmic performance or data modalities, such as imaging and behavioral data, rather than critically evaluating the methodological components underlying robust model development. However, both data augmentation and feature selection are essential for addressing common limitations in ASD datasets, including limited sample sizes, class imbalance, and high‐dimensional feature spaces. Inadequate handling of these challenges may lead to overfitting, reduced generalizability, and misleading performance estimates.

By reviewing how these techniques are implemented and evaluated, this review aims to clarify their contribution to improving predictive performance in ASD research. Furthermore, this review highlights the frequent absence of ablation analyses and pre‐ and post‐comparisons, which makes it difficult to isolate the effects of augmentation or feature selection from those of the underlying model architecture. This lack of methodological transparency limits reproducibility and reduces the clinical applicability of reported findings. Therefore, this review addresses an important gap in the literature by focusing on the practical impact, evaluation, and methodological assessment of these foundational components of ML/DL pipelines for ASD prediction.

## 2 Methods

The study selection process is illustrated in Figure 1 and was conducted in accordance with the Preferred Reporting Items for Systematic Reviews and Meta‐Analyses guidelines [19].

**Figure 1:**
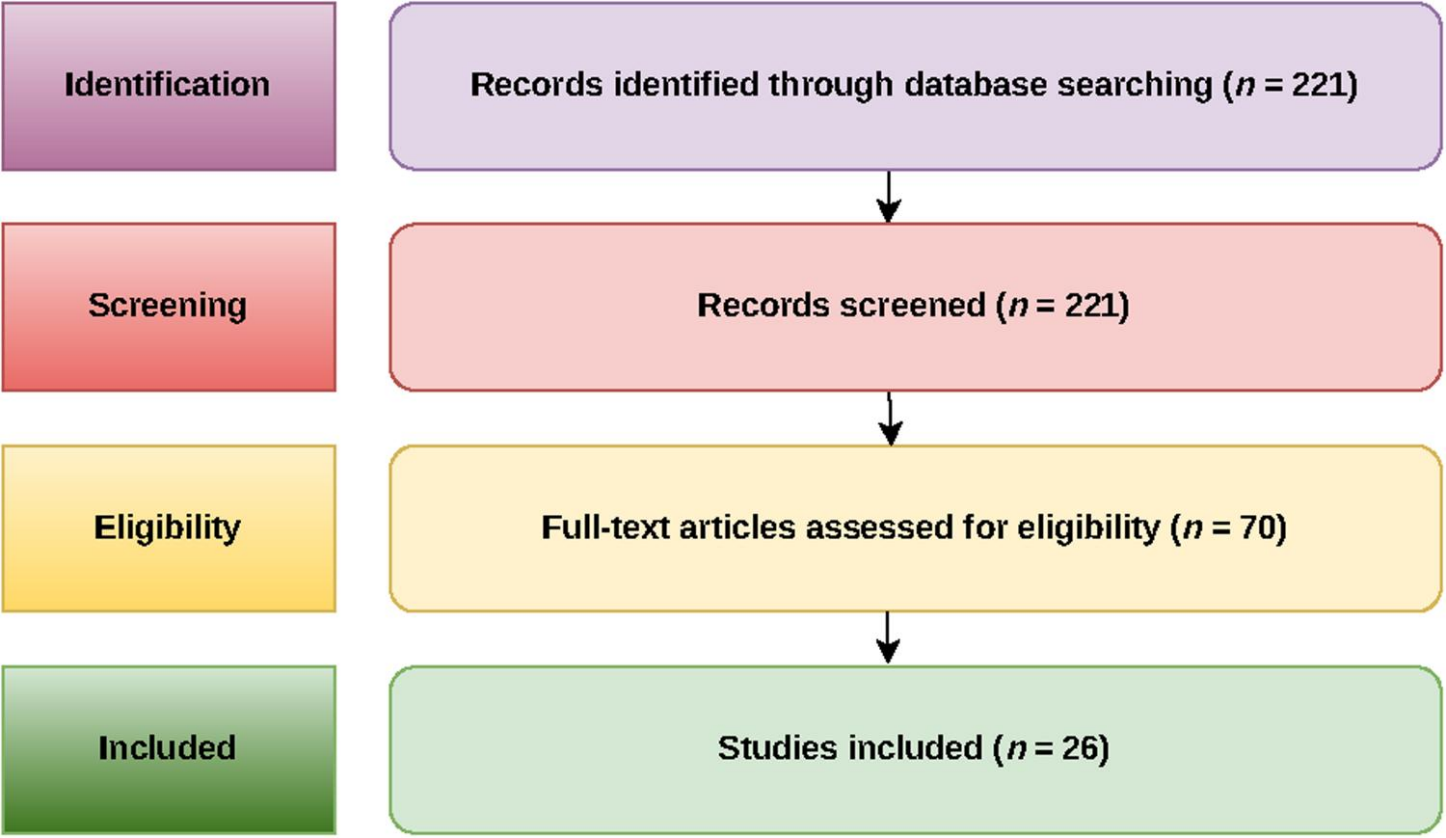
Flowchart of the study identification, screening, eligibility, and inclusion process.

### 2.1 Eligibility Criteria

Titles and abstracts were screened based on the following inclusion criteria: (1) the use of ML or DL techniques for ASD detection; (2) the application of data augmentation and/or feature selection methods; (3) the analysis of human imaging and visual data, including MRI, EEG, eye‐tracking, and facial images; (4) publication in English; and (5) publication between 2021 and 2024.

Studies were excluded if they relied on non‐imaging data modalities, such as genetic data, speech and voice signals, behavioral assessments, blood test results, or hand and head movement data. Segmentation‐focused studies and review articles were also excluded. In addition, studies were further screened to ensure that data augmentation and/or feature selection techniques constituted the primary focus of the research.

The screening process was primarily conducted by the first author. Uncertain cases were resolved through discussion with the supervising author to ensure consistency. Gray literature was excluded from this review. This study was conducted as a narrative review, which does not meet the criteria for PROSPERO registration; therefore, prospective registration was not required.

### 2.2 Search Process, Study Selection, and Result

A review was conducted using six electronic databases: PubMed, Scopus, SpringerLink, IEEE Xplore, ScienceDirect, and Google Scholar. The search covered studies published between 2021 and 2024. However, two studies published in 2020 (Khan et al. [20] and Liu et al. [21]) were retained because of their methodological relevance to the objectives of this review. Specifically, Khan et al. [20] proposed a teacher‐student autoencoder architecture for feature extraction in ASD neuroimaging data, whereas Liu et al. [21] applied Elastic Net regularization as an embedded feature selection method for multi‐site fMRI classification.

Table 1 presents the complete search strategy and the number of records retrieved from each database.

**Table 1:** Search strategy and results across databases.

Database	Search terms	Date range	Records retrieved
PubMed	“machine learning ASD diagnosis” OR “deep learning autism” OR “feature selection ASD” OR “data augmentation autism”	2021–2024	48
Scopus			45
Springer Link			12
IEEE Xplore			54
ScienceDirect			32
Google Scholar			30
Total			221

Abbreviation: ASD, autism spectrum disorder.

A total of 221 records were identified across the six databases. No exact duplicate records were found among the selected databases. Consequently, all 221 records were screened based on their titles and abstracts. Following title and abstract screening, 70 full‐text articles were assessed for eligibility according to the criteria outlined in Section 2.1. Of these, 44 studies were excluded after full‐text assessment. Ultimately, 26 studies focusing on MRI, EEG, eye‐tracking, and facial imaging data were included in this review.

### 2.3 Risk of Bias (ROB) Assessments

The Prediction Model Risk of Bias Assessment Tool (PROBAST) [22, 23] was used to assess the ROB and the applicability of diagnostic and prognostic prediction models included in this review.

PROBAST consists of four domains comprising a total of 20 signaling questions that evaluate ROB and applicability [22, 23]. Each signaling question is rated as “Yes” or “Probably Yes” to indicate low ROB, and “No” or “Probably No” to indicate potential ROB arising from methodological concerns. The “No Information” option is used when insufficient detail is available to determine the appropriate judgment [22, 23].

Responses to the signaling questions inform domain‐level ROB judgments (“Low,” “High,” or “Unclear”), which are subsequently used to derive the overall ROB assessment for each study. A detailed description of the PROBAST domains and signaling questions is provided in the original publication [22, 23].

Before conducting the assessment, the two reviewers discussed the application of the PROBAST tool to ensure a consistent interpretation of the signaling questions. Questions that were not applicable to ML‐based classification studies were rated as “Unclear” according to the PROBAST guidance.

For specific PROBAST items, adapted rating decisions were applied to reflect the characteristics of ML studies. Item 4.7 (performance measures) was rated as “Unclear” when studies reported limited evaluation metrics, such as accuracy alone. Studies that did not report additional performance indicators, including precision, *F*_1_‐score, or the area under the receiver operating characteristic curve (AUC), were considered to provide insufficient evidence for comprehensive model evaluation. In particular, AUC, derived from the receiver operating characteristic curve, provides a more robust assessment of discriminative performance than accuracy alone [17].

Similarly, Items 3.5 (outcome blinding) and 3.6 (time interval) were considered not directly applicable to ML‐based classification studies and were therefore rated as “Unclear.”

The initial bias assessments were conducted independently by the first reviewer and were subsequently reviewed through discussion with the supervising author.

## 3 Overview of ML and DL for ASD Prediction

The diagnosis of ASD can be performed manually or through automated approaches, with ML and DL methods significantly improving diagnostic accuracy across various data sources [6, 24].

Traditional diagnostic approaches primarily rely on clinical observation. In contrast, automated methods employing models such as support vector machines (SVM), random forests (RF), K‐nearest neighbors (KNN), naïve Bayes (NB), and XGBoost have demonstrated promising performance in ASD prediction.

DL frameworks, including convolutional neural networks (CNNs), long short‐term memory networks (LSTMs), generative adversarial networks (GANs), and transfer learning architectures, further enhance predictive capabilities by learning complex hierarchical representations from ASD‐related data. These models particularly benefit from multimodal data that capture the heterogeneous characteristics of ASD.

The data modalities used in ASD research span both medical and non‐medical domains. Medical data include neuroimaging modalities such as MRI, fMRI, and EEG, as well as biological markers including genetic and blood‐based measures. Non‐medical data comprise behavioral assessments, speech signals, hand movement data, and eye‐tracking recordings.

Table 2 presents a structured taxonomy of these modalities as reported in the broader ASD prediction literature. Although the table summarizes all identified data modalities, the present review specifically focuses on imaging and visual data modalities, including MRI, fMRI, sMRI, EEG, eye‐tracking, and facial imaging, in accordance with the eligibility criteria described in Section 2.

**Table 2:** Classification of data types for autism spectrum disorder research.

Category	Data type	Description
Medical data		
	MRI	Neuroimaging techniques such as MRI and PET are frequently used to study the neurodevelopmental traits linked to ASD [25]
	fMRI	fMRI studies primarily analyze the brain's local and global connectivity patterns [25]
	sMRI	sMRI studies commonly use volumetric and morphometric analysis methods to explore any irregularities or abnormalities in the brain's structure [25]
	EEG	Methods for recording functional brain activity like EEG [26]
		A study has indicated distinct EEG patterns in the right and middle parts of the brain in children with ASD compared to typically developing children [27]
	Genetic data	Genetic markers and expression patterns associated with ASD [28]
	Blood test data	Changes observed in numerous proteins in plasma/serum levels of ASD patients, categorized by medications, age, co‐morbid conditions, and ethnicity [29]
Non‐medical data		
	Behavioral assessments	Identifying ASD cases using ten behavioral features (Q‐Chat‐10) and individual characteristics including age, gender, race, jaundice at birth, and family history of ASD
	Speech data	Analysis of prosodic features, energy and zero‐crossing rate, spectral and chroma traits, and MFCCs to detect abnormalities in speech volume, pitch fluctuations, and sound quality [30]
	Hand data	Examination of self‐stimulatory actions including banging the head, flapping the hands, and spinning [31]
	Eye tracking	Evaluation through visual attention, executive function, and cognitive flexibility tasks [32]
	Face images	Analysis of unique facial characteristics in children with autism, including a broader upper face with widely spaced eyes and a relatively shorter central facial region [33]

Abbreviations: ASD, autism spectrum disorder; EEG, electroencephalography; fMRI, functional magnetic resonance imaging; MFCC, Mel‐frequency cepstral coefficient; MRI, magnetic resonance imaging; PET, positron emission tomography; Q‐Chat, Quantitative Checklist for Autism in Toddlers; sMRI, structural MRI.

## 4 Data Augmentation Techniques in ASD Research

Figure 2 classifies the data augmentation techniques identified across the reviewed ASD prediction studies into two main categories: conventional augmentation and generative augmentation methods. The figure provides a concise visual summary of the approaches reported in the reviewed literature.

**Figure 2:**
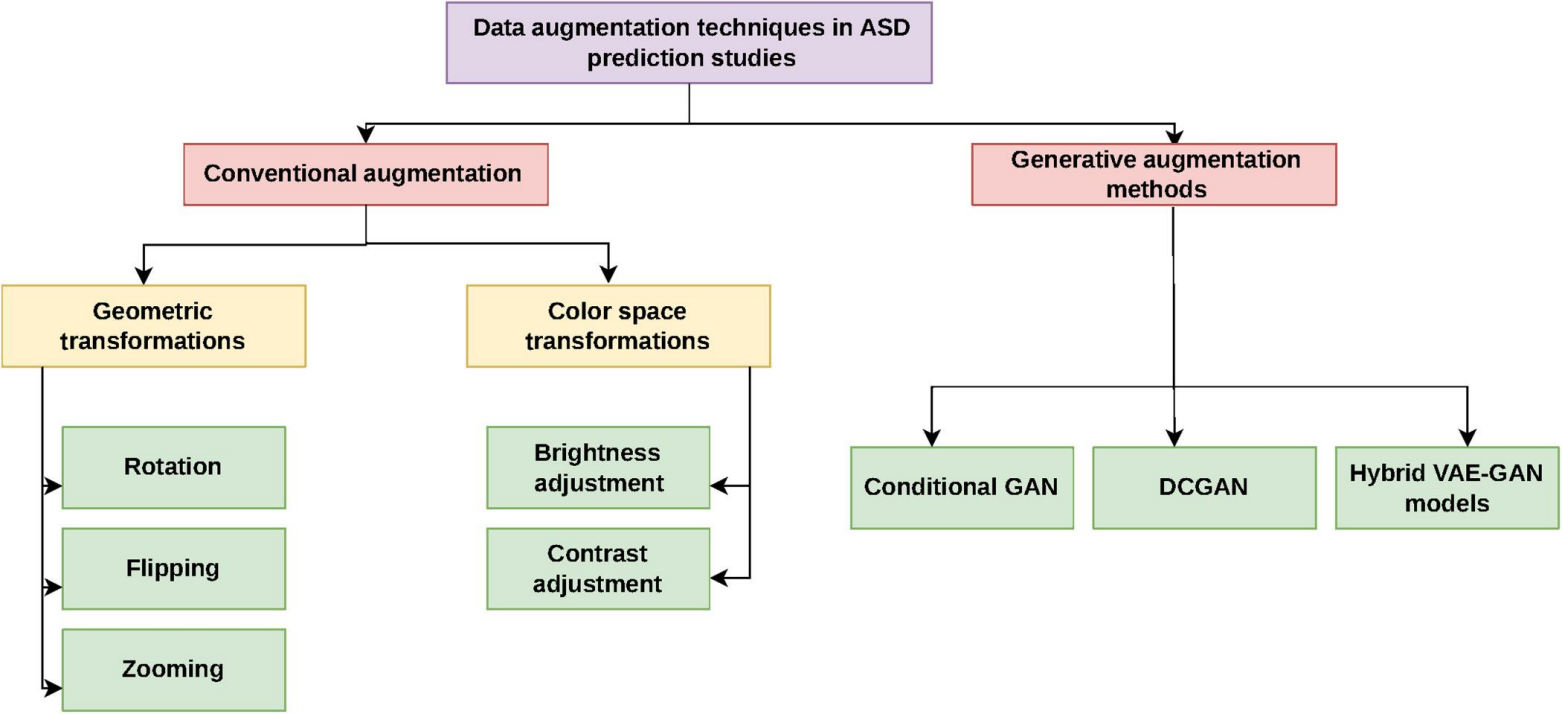
Classification of data augmentation techniques reported in ASD prediction studies included in this review. ASD, autism spectrum disorder; DCGAN, deep convolutional GAN; GAN, generative adversarial network; VAE, variational autoencoder.

Common data augmentation techniques, including geometric transformations, GAN‐based synthesis, and oversampling methods, are widely applied to increase feature diversity and address class imbalance. These approaches can act as regularization strategies, reducing model variance and improving generalizability.

However, the effectiveness of these techniques remains constrained by the limited sample sizes commonly observed in ASD datasets. Therefore, access to large‐scale, standardized, and high‐quality datasets is essential to fully realize the potential of data augmentation methods in ASD prediction [34].

### 4.1 Data Augmentations Based on Basic Image Manipulations

#### 4.1.1 Geometric Transformations

Geometric transformations, including flipping, rotation, translation, zooming, and noise addition, are among the most commonly used augmentation techniques in image‐based ASD studies. Nogay et al. [2] applied horizontal flipping, rotation, and noise augmentation to structural magnetic resonance imaging (sMRI) data, increasing the dataset size fivefold. However, the independent contribution of each augmentation technique was not evaluated, and the reported classification accuracy of 100% raised potential concerns regarding overfitting.

Similarly, Islam et al. [35] augmented eye‐tracking data using rotation, shear, and shift transformations, resulting in a fourfold increase in dataset size. Nevertheless, the study did not separately assess the effect of each augmentation strategy on model performance.

Facial image‐based studies conducted by Khan et al. [36] and Reddy et al. [33] employed pre‐trained CNN architectures, including MobileNetV2, ResNet‐50, and EfficientNetB0, alongside augmentation techniques such as flipping, rotation, zooming, and shifting. Although augmentation appeared to reduce overfitting, neither study included comparative baseline experiments or ablation analyses to quantify the individual contribution of each transformation.

Similarly, Alam et al. [16] combined multiple augmentation techniques on both two‐dimensional and three‐dimensional image data; however, the individual effects of these methods were not isolated. In another study, Alam et al. [17] applied flipping and salt‐and‐pepper noise augmentation, reporting an accuracy of 98.9%. Despite these promising results, the study lacked detailed evaluation and validation procedures.

Overall, basic augmentation techniques may improve model robustness and expand dataset diversity in ASD prediction studies. However, most existing studies do not systematically evaluate the contribution of individual augmentation methods, thereby limiting reproducibility, interpretability, and clinical reliability.

#### 4.1.2 Color Space Transformations

Awaji et al. [37] developed hybrid CNN‐based models, including VGG16, ResNet101, and MobileNet architectures, for ASD detection using facial images. These models were integrated with XGBoost and RF classifiers to improve classification performance. To enhance model training, the authors applied extensive data augmentation techniques, including random rotations, flipping, shifting, zooming, and brightness and contrast adjustments. As a result, the dataset was expanded nearly tenfold, increasing from 2940 to more than 20,000 images.

The final model, which combined RF classification with VGG16‐MobileNet feature extraction, achieved high predictive performance, reporting an AUC of 99.25% and an accuracy of 98.8%. However, the study did not include baseline comparisons or ablation analyses to evaluate the specific contribution of augmentation strategies to the reported performance improvements.

Although color‐space and geometric augmentation techniques improved dataset diversity, these transformations may not fully capture real‐world facial variability. In addition, the reliance on a relatively small original dataset may limit the generalizability of the proposed model. Furthermore, the absence of comparative evaluation between augmented and non‐augmented datasets makes it difficult to determine whether the observed performance gains were primarily attributable to the model architecture or the augmentation procedures. This limitation reduces the reproducibility, interpretability, and clinical reliability of the reported findings.

### 4.2 Data Augmentations Based on DL

GANs are widely used for synthetic data generation by learning underlying distributions from real datasets. In ASD research, GAN‐based augmentation approaches have been applied to address dataset scarcity and class imbalance.

Raja et al. [38] employed conditional GANs to augment functional magnetic resonance imaging (fMRI) data for ASD prediction. Although the proposed approach improved classification performance, the study did not incorporate time‐series augmentation or comparative baseline evaluations, limiting the interpretability of the reported improvements.

Similarly, Xu et al. [26] combined traditional augmentation techniques, including flipping and cropping, with deep convolutional GAN‐generated EEG data within a CNN‐LSTM framework. The proposed model achieved accuracies of 81.08% and 74.55% for resting‐state and task‐state EEG data, respectively. However, the independent contribution of each augmentation strategy was not separately evaluated.

Lalli et al. [39] proposed DEBCWGAN, a hybrid variational autoencoder‐weighted GAN framework for EEG augmentation, and integrated it with the Rest‐HGCN model. The proposed approach achieved a classification accuracy of 91.67% while addressing class imbalance issues. Although the study demonstrated improved performance compared with models without augmentation, further comparative analyses across different GAN architectures and augmentation strategies remain necessary.

Overall, GAN‐based augmentation techniques demonstrate considerable potential for improving ASD prediction performance. However, their effectiveness largely depends on the quality, diversity, and realism of the generated samples, as well as the use of rigorous evaluation protocols [26, 39].

Notably, two studies combined GAN‐based synthetic generation with conventional geometric augmentation techniques. Xu et al. [26] integrated deep convolutional GAN‐generated EEG data with cropping, flipping, and Gaussian noise augmentation, whereas Raja et al. [38] combined conditional GAN‐based synthesis with geometric transformations, including flipping and rotation, for fMRI data augmentation. Although this hybrid strategy may leverage the complementary strengths of conventional and GAN‐based augmentation methods, neither study isolated the independent contribution of each augmentation component through ablation analyses or comparative evaluation.

### 4.3 Summary of Data Augmentation Techniques

Data augmentation has been associated with reported improvements in model performance and generalizability in ASD prediction, primarily by increasing dataset size and enhancing training diversity, as demonstrated across the studies summarized in Table 3.

**Table 3:** Quantitative comparison of data augmentation techniques and evaluation strategies in ASD studies.

Study	Category	Dataset type	Sample size (*n*)	Accuracy (%)	Ablation	Validation
Xu et al. [26]	Generative + traditional (Sections 4.1.1 and 4.2)	EEG	189	81.08 (Resting) 74.55 (Task)	No	5‐fold CV only
Lalli et al. [39]	Generative (DEBCWGAN) (Section 4.2)	EEG	56	91.67 (EEG‐ASD) 88.10 (ABC‐CT)	No	Hold‐out only
Nogay et al. [2]	Traditional (rotation, mirroring, noise) (Section 4.1.1)	sMRI	1831	100 (Acc + Recall)	No	5‐fold CV only
Raja et al. [38]	Generative + traditional (cGAN, flips, rotations) (Sections 4.1.1 and 4.2)	fMRI	2226	74 (Acc) 79 (Sen) 64 (Spe)	No	10‐fold CV only
Islam et al. [35]	Traditional (rotation, shear, width shift) (Section 4.1.1)	Eye‐tracking	547 300	99.43 (D1) 96.78 (D2)	No	Hold‐out only
Khan et al. [36]	Traditional (horizontal flip, rotation, norm) (Section 4.1.1)	Facial images	2940	96 (Acc) 96 (Pre + Rec + *F*_1_)	No	Hold‐out only
Alam et al. [16]	Traditional (rotation, zoom, flip, shear) (Section 4.1.1)	Facial images	40	100 (2D) 93.75 (3D)	No	Hold‐out only
Reddy et al. [33]	Traditional (rotation, flip, zoom, shift) (Section 4.1.1)	Facial images	3014	87.9 (EffNet) 84.66 (VGG16)	No	Hold‐out only
Alam et al. [17]	Traditional + pre‐processing (Section 4.1.1)	Facial images	3014	98.9 (Acc) 99.9 (AUC)	No	Hold‐out only
Awaji et al. [37]	Traditional + *t*‐SNE (Section 4.1.2)	Facial images	2940	98.8 (Acc) 99.25 (AUC)	No	5‐fold CV + hold‐out

Abbreviations: 2D, two‐dimensional; 3D, three‐dimensional; ABC‐CT, Autism Biomarkers Consortium for Clinical Trials; Acc, accuracy; ASD, autism spectrum disorder; AUC, area under the receiver operating characteristic curve; cGAN, conditional generative adversarial network; CV, cross‐validation; DEBCWGAN, dual‐encoder based conditional Wasserstein generative adversarial network; EEG, electroencephalography; EffNet, EfficientNet; fMRI, functional magnetic resonance imaging; Pre, precision; Rec, recall; Sen, sensitivity; sMRI, structural magnetic resonance imaging; Spe, specificity; *t*‐SNE, *t*‐distributed Stochastic Neighbor Embedding; VGG16, Visual Geometry Group 16‐layer network.

However, the strength of this evidence remains limited. As indicated in Table 4, only a small proportion of studies conducted ablation analyses or applied external validation procedures, making it difficult to isolate the contribution of individual augmentation techniques. This lack of methodological rigor reduces confidence in attributing reported performance improvements directly to the applied augmentation methods.

**Table 4:** Distribution of data augmentation techniques, ablation studies, and validation methods in ASD literature.

Category	Method	Studies (*n*)	Dataset type	Sample size range	Best accuracy range (%)	Ablation (*n*)	External validation (*n*)
Geometric transformations (*n* = 6)	Rotation, flip, zoom, shear	5	sMRI, eye‐tracking, facial	40–3014	84.66–100.00	0/5	0/5
	Geometric + pre‐processing (Gaussian, noise)	1	Facial	3014	98.90	0/1	0/1
Color space transformations (*n* = 1)	Brightness/contrast + *t*‐SNE	1	Facial	2940	98.80	0/1	0/1
Generative (*n* = 1)	GAN‐based (cGAN, DEBCWGAN)	1	EEG, fMRI	56	91.67	0/1	0/1
Geometric + generative (*n* = 2)	GAN + flips/rotations	2	EEG, fMRI	189–2226	74.00–81.08	0/2	0/2

Abbreviations: ASD, autism spectrum disorder; cGAN, conditional generative adversarial network; DEBCWGAN, dual‐encoder based conditional Wasserstein generative adversarial network; EEG, electroencephalography; fMRI, functional magnetic resonance imaging; GAN, generative adversarial network; *n*, number; sMRI, structural magnetic resonance imaging; *t*‐SNE, *t*‐distributed Stochastic Neighbor Embedding.

Among conventional augmentation strategies, geometric transformations such as flipping, rotation, and shifting, together with color space adjustments including brightness and contrast modifications, were the most frequently reported techniques, as summarized in Table 4. Although these approaches may help reduce overfitting in DL models using pre‐trained CNNs, the absence of systematic evaluation frameworks limits reproducibility and restricts accurate assessment of their individual contributions.

More advanced DL‐based augmentation approaches, particularly those based on GANs, have shown considerable potential for addressing dataset scarcity and class imbalance. Studies using GAN‐generated synthetic fMRI and EEG data have reported improved classification performance, with some studies achieving accuracies of up to 91.67%, while also suggesting improved learning of minority class distributions. Despite these promising findings, several limitations remain. Few studies evaluate the quality, diversity, or realism of generated samples, and comparative analyses across different GAN architectures remain limited. In addition, temporal characteristics, which are particularly important for sequential modalities such as fMRI and EEG, are frequently overlooked in GAN training pipelines.

In summary, although both conventional and GAN‐based augmentation techniques demonstrate potential for improving ASD prediction models, important methodological limitations remain. These include insufficient baseline comparisons between original and augmented datasets, limited use of external validation, inadequate ablation analyses, and insufficient integration of temporal information in neural data. Addressing these limitations is essential for improving the robustness, reproducibility, interpretability, and clinical applicability of ASD prediction models.

## 5 Feature Selection Techniques

Feature selection plays a crucial role in improving model performance, interpretability, and computational efficiency in ML, particularly in high‐dimensional tasks such as ASD prediction. By identifying the most informative features, feature selection helps mitigate overfitting, simplify model complexity, and improve model generalizability. Feature selection methods are generally categorized into three main groups: filter, wrapper, and embedded approaches, each employing distinct evaluation strategies.

Figure 3 classifies the feature selection techniques identified across the reviewed ASD prediction studies into three categories: filter‐based, wrapper‐based, and embedded methods, providing a visual summary of the approaches reported in the literature.

**Figure 3:**
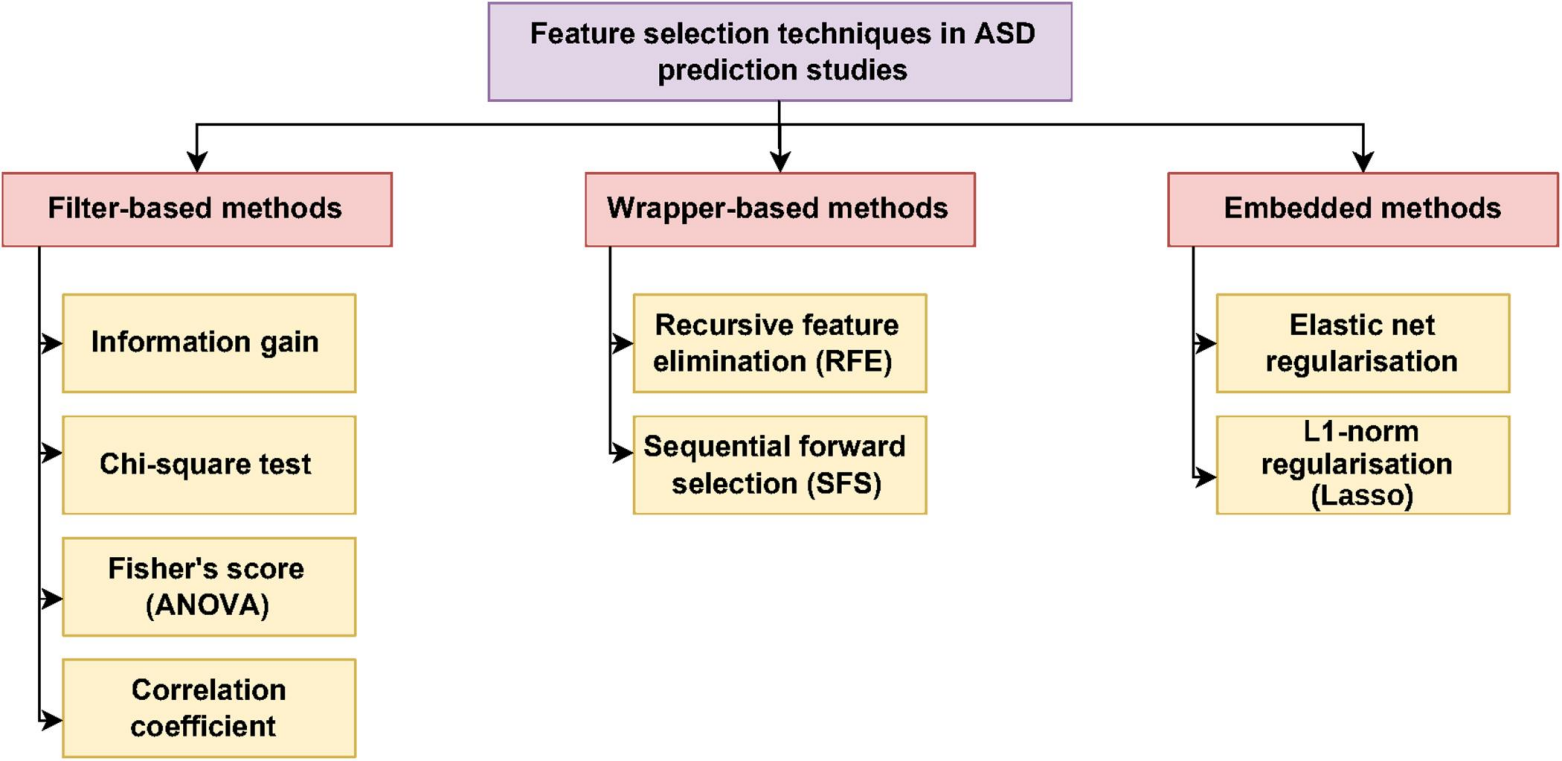
Classification of feature selection techniques reported in ASD prediction studies included in this review. ASD, autism spectrum disorder; ANOVA, Analysis of Variance; RFE, recursive feature elimination; SFS, sequential forward selection.

### 5.1 Filter‐Based Feature Selection Methods

Filter‐based feature selection methods rank features statistically before model training using either univariate or multivariate approaches. Univariate methods evaluate each feature independently, whereas multivariate methods consider the joint relevance and interaction among features [40]. These approaches are computationally efficient and help reduce irrelevant or redundant features; however, their effectiveness depends heavily on the underlying data structure and the selected scoring metrics. The following filter‐based methods were identified in ASD prediction studies:

(1) Information gain: Ahmed et al. [41] used RF‐based ranking to evaluate eye‐tracking features according to information gain, resulting in improved model performance. The proposed LSTM and CNN‐LSTM models achieved classification accuracies of up to 98.33%. However, the study did not investigate how the number of selected features influenced model performance, raising concerns regarding overfitting and reproducibility.

(2) Chi‐square test: Xu et al. [26] applied the SelectKBest algorithm with the chi‐square test to EEG‐derived features following Pearson correlation‐based connectivity mapping. Although this approach improved interpretability by identifying potentially relevant brain regions, the study did not justify the selection of the *K* parameter or evaluate performance variations across different feature subset sizes.

(3) Fisher's score (ANOVA *F*‐test): Ladani et al. [42] employed principal component analysis (PCA) followed by the ANOVA *F*‐score method to reduce the dimensionality of fMRI data from 800 to 600 features. Although this approach aimed to reduce computational cost and mitigate overfitting, the linear assumptions underlying PCA and ANOVA may overlook complex non‐linear relationships within the data. Notably, models without dimensionality reduction achieved superior performance, raising questions regarding the effectiveness of the applied feature selection strategy.

(4) Correlation coefficient: Lylath et al. [25] used Pearson correlation to construct statistical dependency networks from MRI data and applied rank correlation coefficients to identify relevant features. Although this method improved computational efficiency, it remained sensitive to outliers and lacked quantitative evaluation of its impact on classification performance. Furthermore, the number of selected features was not adequately justified, limiting interpretability and reproducibility.

### 5.2 Wrapper‐Based Feature Selection Methods

Wrapper‐based feature selection methods evaluate feature subsets by repeatedly training predictive models to maximize classification performance. Unlike filter‐based approaches, wrapper methods consider interactions among features, which may improve predictive accuracy but at the cost of increased computational complexity. These methods are particularly relevant in ASD research because of the high dimensionality and limited sample sizes commonly associated with neuroimaging and behavioral datasets. However, many studies do not adequately justify feature subset sizes or provide comparative baseline evaluations.

(1) Recursive feature elimination (RFE): Bahathiq et al. [43] evaluated several wrapper‐based feature selection approaches, including RFE with cross‐validation (RFECV), Boruta, and the Grey Wolf Optimizer (GWO), for dimensionality reduction in ASD prediction. RFECV iteratively removes the least informative features, whereas Boruta compares original features with randomly generated shadow features using *Z*‐score‐based importance measures. GWO applies a heuristic optimization strategy to identify optimal feature subsets. These approaches reduced feature redundancy and computational cost while maintaining predictive performance. However, the study lacked methodological transparency regarding feature ranking and selection procedures. In addition, the use of a relatively small dataset increased the risk of overfitting and limited the external validity of the findings. The absence of detailed methodological reporting also reduced reproducibility.

(2) Sequential forward feature selection (SFFS): Khan et al. [20] implemented SFFS within a three‐stage pipeline using resting‐state functional magnetic resonance imaging (rs‐fMRI) data. Features were incrementally added according to their contribution to classifier accuracy. Although the method effectively removed irrelevant features, its high computational cost may limit scalability for large datasets. Furthermore, several methodological decisions, including the addition of 50 features per iteration, were not adequately justified and may have prevented identification of optimal feature subsets. The study also lacked comparative analyses between models with and without feature selection and did not interpret the selected features within a clinical or neurobiological context, thereby limiting interpretability and clinical relevance.

### 5.3 Embedded Feature Selection Methods

Embedded feature selection methods integrate feature selection directly into the model training process, combining the computational efficiency of filter‐based methods with the predictive advantages of wrapper‐based approaches. These methods commonly employ regularization techniques, such as Lasso, Ridge, and Elastic Net, to promote feature sparsity [40]. Consequently, embedded methods are particularly suitable for high‐dimensional ASD datasets, including EEG and fMRI data, where model interpretability and generalizability are essential.

(1) Elastic Net regularization: Liu et al. [21] applied Elastic Net regularization to rs‐fMRI data for ASD classification. Elastic Net combines L1 (Lasso) and L2 (Ridge) regularization penalties to improve robustness and reduce overfitting. Although the proposed approach outperformed both Ridge and Lasso individually, the study did not provide comparative evaluations of model performance before and after feature selection. In addition, details regarding hyperparameter optimization were not reported, thereby limiting reproducibility and methodological transparency.

(2) L1‐norm regularization (Lasso): Peng et al. [44] used L1‐norm regularization to select features from EEG data collected under emotional stimuli. The proposed model achieved classification accuracies of up to 93.8%, benefiting from reduced feature dimensionality and increased sparsity. However, L1‐based regularization may introduce coefficient shrinkage bias, potentially affecting feature interpretability. Furthermore, the study did not evaluate the influence of feature subset size on predictive performance. The absence of cross‐validation procedures and insufficient justification of the selected feature subset reduce confidence in the generalizability and reproducibility of the reported findings.

### 5.4 Summary of Feature Selection Methods

As summarized in Table 5, the feature selection techniques identified across the included studies can be categorized into filter‐based, wrapper‐based, and embedded methods. Among these approaches, filter‐based methods were the most frequently applied, followed by wrapper and embedded techniques.

**Table 5:** Distribution of feature selection techniques, ablation studies, and validation practices in ASD research.

Category	Method	Studies (*n*)	Dataset used	Sample size	Best accuracy (%)	Ablation study	External validation
Filter‐based (*n* = 4)	Information gain	1	Eye‐tracking	59	98.33	No	No
	Chi‐square test	1	EEG	189	81.08	No	No
	Fisher's score (ANOVA)	1	fMRI	1035	71.24	No	No
	Correlation coefficient	1	fMRI	1102	83.46	No	No
Wrapper‐based (*n* = 2)	RFE (RFECV, Boruta, GWO)	1	sMRI	671	71.00	No	Yes
	SFFS	1	rs‐fMRI	1035	82.00	No	No
Embedded (*n* = 2)	E Net	1	rs‐fMRI	468	83.33	No	No
	LASSO (L1‐norm)	1	EEG	64	93.80	No	No

Abbreviations: ANOVA, analysis of variance; ASD, autism spectrum disorder; E‐Net, Elastic Net; EEG, electroencephalography; fMRI, functional magnetic resonance imaging; GWO, Grey Wolf Optimizer; Lasso, least absolute shrinkage and selection operator; RFE, recursive feature elimination; RFECV, recursive feature elimination with cross‐validation; rs‐fMRI, resting‐state functional magnetic resonance imaging; SFFS, sequential forward feature selection; sMRI, structural magnetic resonance imaging.

However, only a limited number of studies conducted rigorous validation procedures, such as ablation analyses or external validation. None of the included studies explicitly evaluated the independent contribution of feature selection through systematic ablation analysis. Similarly, only a small proportion of studies employed external validation strategies. These findings indicate that, although feature selection is widely used in ASD prediction, its impact is rarely assessed in a rigorous and reproducible manner.

Feature selection represents a critical component for improving the accuracy, generalizability, interpretability, and computational efficiency of ML models for ASD detection. This is particularly important when analyzing high‐dimensional datasets, such as EEG and fMRI data, where large numbers of irrelevant or redundant features may negatively affect predictive performance and model interpretability.

Filter methods, including information gain, chi‐square tests, ANOVA *F*‐score, and correlation analysis, are widely used because of their simplicity and computational efficiency. However, these methods often fail to capture dependencies among features. Wrapper methods, such as RFE and SFFS, may provide improved predictive performance by evaluating feature subsets based on model feedback. Nevertheless, these approaches are computationally intensive and may have limited scalability. Embedded methods, including Lasso, Ridge, and Elastic Net regularization, integrate feature selection directly into the model training process, thereby balancing feature sparsity and predictive performance.

Despite their potential benefits, current studies applying feature selection in ASD prediction frequently exhibit important methodological limitations. Few studies perform rigorous validation procedures, including cross‐validation, pre‐ and post‐comparisons, or ablation analyses, to quantify the specific contribution of feature selection to predictive performance. In addition, the number of selected features is often determined arbitrarily without empirical optimization or sensitivity analysis, raising concerns regarding overfitting and reproducibility.

Importantly, many studies do not interpret selected features within clinical or neurobiological contexts, thereby limiting their applicability to real‐world diagnostic settings and reducing transparency for healthcare professionals.

To fully realize the potential of feature selection in ASD‐related ML applications, future research should emphasize methodological transparency, robust evaluation frameworks, and domain‐informed interpretation of selected features. Such improvements would strengthen both the scientific credibility and clinical applicability of predictive models in ASD research.

## 6 Application of ML and DL Models in ASD Prediction

ML and DL models play an important role in ASD research by extracting informative patterns from complex data, thereby supporting early diagnosis and automated prediction.

These models span classical approaches, including supervised methods such as SVM and unsupervised methods such as PCA, as well as DL architectures such as CNNs and recurrent neural networks (RNNs), each contributing to pattern detection, sequence modeling, and generative data synthesis.

This diversity of approaches contextualizes the transition from traditional ML to DL methods better suited to complex biomedical data. The following sections review recent applications, strengths, limitations, and deployment challenges of these models in ASD prediction.

### 6.1 Traditional ML Models

Several traditional ML models, including SVM, RF, NB, logistic regression, and KNN, have been applied to ASD prediction using behavioral and neuroimaging features [45]. These approaches have demonstrated promising performance across various ASD‐related classification tasks because of their relative interpretability and computational efficiency.

For example, Ridge classifiers applied to wavelet‐transformed fMRI data achieved promising predictive performance [42]. However, the linear nature of Ridge‐based models and their reliance on phenotypic variables, such as age and gender, may limit their clinical applicability. Similarly, a linear SVM model combined with L1 regularization improved classification performance using EEG signals; nevertheless, the study was conducted using a relatively small sample size, increasing the risk of overfitting and limiting generalizability [44].

Eye‐tracking data have also shown potential for early ASD detection, with some ML models reporting AUC values approaching 0.85 [32]. Despite these encouraging findings, many studies continue to rely on small and imbalanced datasets and rarely incorporate external validation procedures, raising concerns regarding the robustness and generalizability of the reported models.

Overall, although traditional ML approaches offer advantages in interpretability and implementation simplicity, they often struggle to capture the complex non‐linear and high‐dimensional patterns associated with ASD. These limitations have motivated increasing interest in DL‐based approaches for ASD prediction.

### 6.2 DL Models for ASD Prediction

DL models have demonstrated strong potential in ASD prediction by effectively capturing spatial, temporal, and behavioral patterns from EEG, neuroimaging, and eye‐tracking data. Architectures such as CNNs, RNNs, LSTM networks, and GANs frequently outperform traditional ML models in predictive performance. However, several limitations remain, including small dataset sizes, overfitting, insufficient validation procedures, and limited model interpretability. The following section reviews recent applications of DL models in ASD prediction.

(1) Artificial neural networks: Mezrioui et al. [34] developed an artificial neural network model combining sMRI and fMRI data to characterize ASD across developmental stages. The model effectively captured structural and functional brain features, but its clinical utility was restricted by a small sample size (521 cases) and interpretability challenges.

(2) CNNs: CNN‐based models have demonstrated high classification performance in ASD prediction using eye‐tracking and neuroimaging data [2, 46]. In particular, the hybrid Rest‐HGCN framework achieved improved EEG‐based classification performance, reporting accuracies ranging from 87.1% to 91.6% [27, 39]. However, several limitations remain, including limited external validation, dataset homogeneity, and limited model interpretability, all of which restrict clinical translation. Although GAN‐augmented datasets helped mitigate class imbalance, challenges related to black‐box decision‐making and limited dataset diversity persist.

(3) RNNs and LSTMs: Xu et al. [26] proposed a CNN‐LSTM framework for EEG‐derived brain connectivity mapping that combined spatial and temporal feature learning. Similarly, Ahmed et al. [41] evaluated LSTM, CNN‐LSTM, gated recurrent unit, and Bidirectional LSTM models using statistical eye‐tracking data, achieving classification accuracies of up to 98.33%. Despite these promising results, the models were constrained by relatively small sample sizes, lack of external validation, high computational complexity, and limited interpretability. These limitations reduce clinical trust and hinder real‐time deployment. Future research may benefit from lightweight transfer learning architectures, such as MobileNet‐based frameworks with fine‐tuning strategies, to reduce computational overhead while maintaining predictive performance.

(4) GANs: Raja et al. [38] introduced a conditional GAN framework to generate synthetic fMRI data for ASD classification, reporting improved predictive performance across multi‐site datasets. However, the model relied exclusively on fMRI data and demonstrated high sensitivity (79%) but comparatively lower specificity (64%), raising concerns regarding false‐positive predictions and potential over‐diagnosis. Although the proposed approach demonstrated methodological novelty, the absence of multimodal integration and limited specificity reduce confidence in its real‐world clinical applicability and reliability.

### 6.3 Transfer Learning and Hybrid Models

Transfer learning and hybrid modeling approaches have recently gained considerable attention in ASD prediction, particularly for addressing challenges associated with small and imbalanced datasets. Transfer learning enables knowledge transfer from large annotated source domains to target domains with limited data availability, thereby reducing training time, improving model convergence, and facilitating the reuse of pre‐trained architectures for ASD detection.

Hybrid modeling approaches combine DL techniques for feature extraction with classical ML algorithms for classification. These approaches aim to balance predictive performance, interpretability, and computational efficiency. However, challenges such as overfitting and insufficient validation procedures remain significant limitations.

(1) Transfer learning: Khan et al. [36] employed pre‐trained CNN architectures, including MobileNetV2, Xception, and ResNet‐50, for ASD classification using facial image data. Similarly, Reddy et al. [33] reported high AUC values using EfficientNetB0, while other studies utilized architectures such as ResNet50V2, AlexNet, and hybrid CNN‐based models with promising classification performance [16, 25, 47]. However, many of these findings were derived from relatively small and non‐clinical datasets and lacked external validation, thereby limiting real‐world applicability and generalizability. Transfer learning approaches have also been investigated in eye‐tracking and retinal imaging studies [48, 49], although similar methodological limitations remain. While transfer learning may improve learning efficiency and predictive accuracy, challenges related to model interpretability, overfitting, and clinical validation continue to limit its clinical translation.

(2) Hybrid approaches: Studies integrating DL‐based feature extraction with traditional ML classifiers have demonstrated promising predictive performance. For example, Manoj et al. [7] combined RF classifiers with VGG16‐ and MobileNet‐derived features, achieving classification accuracies of up to 99.25%. Similarly, EEG‐based hybrid models, including Rest‐HGCN and CNN‐LSTM architectures, outperformed several traditional baseline models [26]. Multi‐stage and integrated frameworks using pre‐trained CNNs for facial image analysis also reported improved diagnostic performance [20, 37, 50]. Despite these advancements, most proposed models were developed using limited datasets and lacked comprehensive external validation. Consequently, concerns regarding overfitting, model generalizability, and deployment complexity remain significant barriers to clinical implementation.

## 7 Quality Assessment and ROB

The detailed PROBAST assessment is presented in Table 6, while a visual summary is provided in Figure 4.

**Table 6:** ROB assessment of included studies using the PROBAST tool.

	**D1**		D1 ROB	**D2**			D2 ROB	**D3**						D3 ROB	**D4**									D4 ROB	Overall ROB
**Study**	1.1 Data source	1.2 Inclusion/exclusion criteria		2.1 Predictor standardization	2.2 Predictor blinding	2.3 Predictor availability		3.1 Outcome misclassification	3.2 Prespecification	3.3 Incorporation	3.4 Outcome standardization	3.5 Outcome blinding	3.6 Time interval		4.1 Sample size	4.2 Handling predictors	4.3 Missing data	4.4 Missing data handling	4.5 Univariable selection	4.6 Data complexity	4.7 Performance measures	4.8 Overfitting	4.9 Predictor weights		
Lylath et al. [25]	L	U	U	U	U	L	U	L	L	L	U	L	U	U	L	U	U	U	H	U	U	U	L	H	H
Ladani et al. [42]	L	U	U	L	U	L	U	L	L	L	U	L	U	U	L	L	U	U	H	U	U	U	L	H	H
Bahathiq et al. [43]	L	L	L	L	U	L	U	L	L	L	L	L	U	U	L	L	L	L	L	U	U	U	L	U	U
Nogay et al. [2]	L	U	U	U	U	L	U	L	L	L	U	L	U	U	L	U	U	U	L	U	U	U	L	U	U
Raja et al. [38]	L	U	U	U	U	L	U	L	L	L	U	L	U	U	L	U	U	U	L	U	L	U	L	U	U
Khan et al. [20]	L	L	L	L	U	L	U	L	L	L	L	L	U	U	L	L	U	U	L	U	U	U	L	U	U
Liu et al. [21]	L	U	U	U	U	L	U	L	L	L	U	L	U	U	L	U	U	U	L	U	U	U	L	U	U
Xu et al. [26]	L	U	U	U	U	L	U	L	L	L	U	L	U	U	H	U	U	U	H	U	U	U	L	H	H
Menaka et al. [51]	L	U	U	L	U	L	U	L	L	L	L	L	U	U	H	L	U	U	L	U	U	U	L	H	H
Tang et al. [27]	L	U	U	L	U	L	U	L	L	L	U	L	U	U	L	L	U	U	L	U	U	U	L	U	U
Lalli et al. [39]	L	U	U	L	U	L	U	L	L	L	U	L	U	U	H	L	U	U	L	U	U	U	L	H	H
Peng et al. [44]	L	L	L	L	U	L	U	L	L	L	L	L	U	U	H	L	U	U	H	U	H	U	L	H	H
Wei et al. [32]	L	U	U	L	U	L	U	L	L	L	L	L	U	U	H	L	L	L	L	U	L	U	L	H	H
Alsaidi et al. [46]	L	U	U	U	U	L	U	L	L	L	L	L	U	U	H	U	U	U	L	U	U	U	L	H	H
Islam et al. [35]	L	U	U	U	U	L	U	L	L	L	U	L	U	U	U	U	U	U	L	U	U	U	L	U	U
Thanarajan et al. [49]	U	U	U	U	U	L	U	L	L	L	H	L	U	H	H	H	U	U	L	U	U	U	L	H	H
Kim et al. [48]	L	L	L	U	U	L	U	L	L	L	L	L	U	U	L	U	U	U	L	U	U	U	L	U	U
Ahmed et al. [41]	L	U	L	U	U	L	U	L	L	L	U	L	U	U	H	U	U	U	L	U	L	U	L	H	H
Khan et al. [36]	U	H	H	L	U	L	U	L	L	L	H	L	U	H	U	L	U	U	L	U	L	U	L	U	H
Reddy et al. [33]	U	H	H	L	U	L	U	L	L	L	H	L	U	H	U	L	L	L	L	U	U	U	L	U	H
Darmiyati et al. [52]	L	U	U	L	U	L	U	L	L	L	L	L	U	U	U	L	U	U	L	U	U	U	L	U	U
Varsha et al. [53]	U	U	U	U	U	L	U	L	L	L	H	L	U	H	U	U	U	U	L	U	U	U	L	U	H
Alam et al. [17]	U	U	U	L	U	L	U	L	L	L	H	L	U	H	U	L	U	U	L	U	L	U	L	U	H
Alam et al. [16]	L	U	U	L	U	L	U	L	L	L	H	L	U	H	H	L	U	U	L	U	L	U	L	H	H
Li et al. [50]	U	U	U	L	U	L	U	L	L	L	H	L	U	H	U	L	U	U	L	U	L	U	L	U	H
Awaji [37]	U	U	U	L	U	L	U	L	L	L	H	L	U	H	U	L	U	U	L	U	L	U	L	U	H

*Note:* Red indicates high risk of bias (H), green indicates low risk of bias (L), and yellow indicates unclear risk of bias (U).

Abbreviations: D1, domain 1; D2, domain 2; D3, domain 3; D4, domain 4; H, high risk of bias; L, low risk of bias; PROBAST, Prediction Model Risk of Bias Assessment Tool; ROB, risk of bias; U, unclear risk of bias.

**Figure 4:**
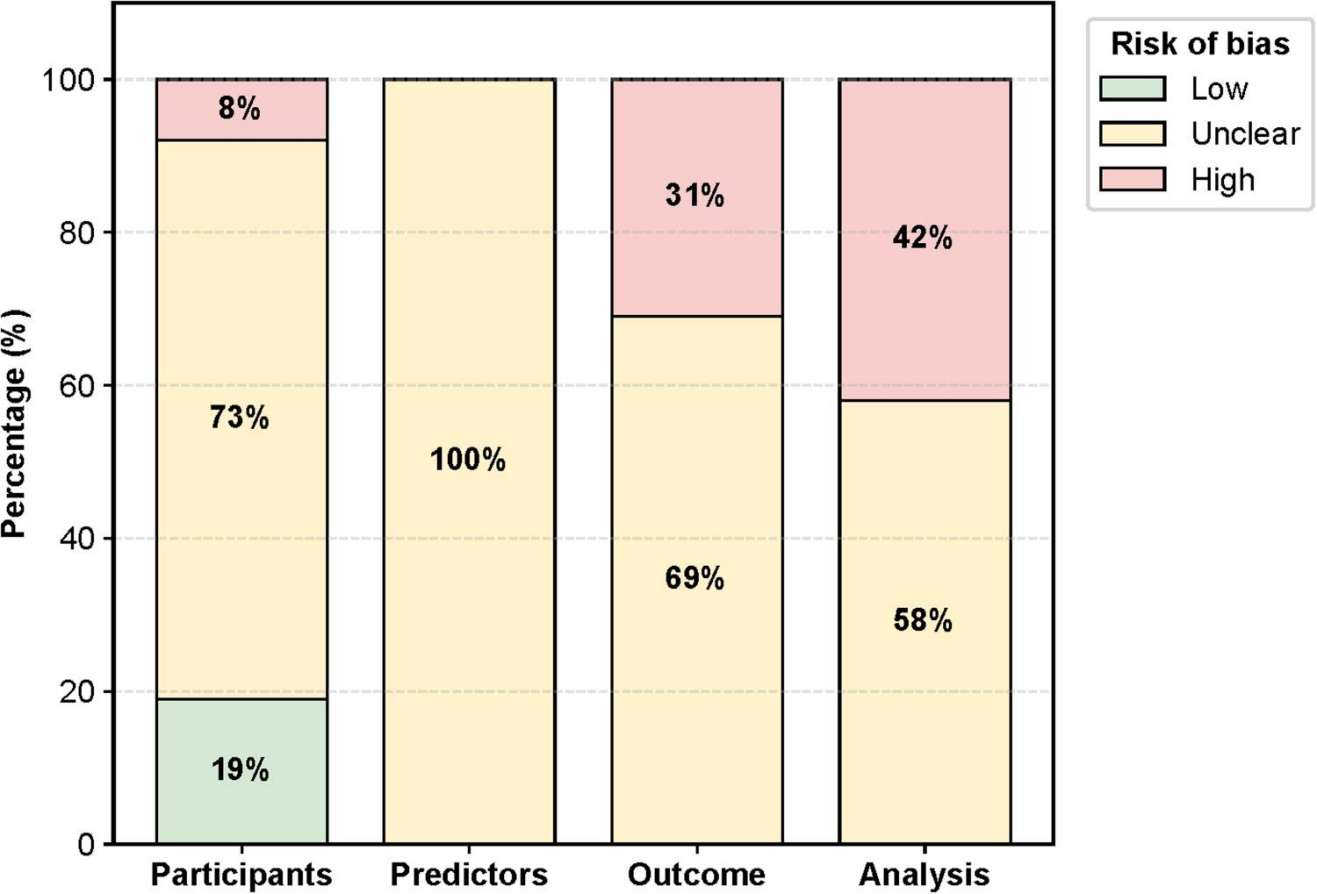
Summary of risk of bias by domain using the PROBAST tool across included studies (*n* = 26). PROBAST, Prediction Model Risk of Bias Assessment Tool.

Overall, the 26 included studies demonstrated a predominantly unclear risk‐of‐bias profile, primarily reflecting insufficient methodological reporting rather than confirmed methodological deficiencies.

As illustrated in Figure 4, the Analysis domain (D4) exhibited the highest proportion of high‐risk assessments (42%). This finding was mainly associated with small sample sizes and the reliance on univariable feature selection methods. In addition, 58% of studies were classified as having an unclear risk due to insufficient reporting of missing data handling procedures. Many studies reported limited performance metrics, often relying solely on accuracy or recall without including AUC, precision, or *F*_1_‐score measures. Furthermore, evidence regarding strategies to mitigate overfitting was frequently insufficiently reported.

The Outcome domain (D3) showed 31% high ROB, largely attributable to inadequate standardization of outcome assessment. Several studies did not report consistent application of DSM‐5 diagnostic criteria, raising concerns regarding outcome standardization.

In contrast, the Predictors domain (D2) demonstrated 100% unclear ROB. This result was substantially influenced by Item 2.2 (predictor blinding), which is not directly applicable to ML classification studies because predictor blinding is generally irrelevant in this context. Since the standard PROBAST tool does not provide a “not applicable” option for this item, an unclear rating was assigned. Consequently, the predominance of unclear ratings in this domain may reflect limitations of the PROBAST framework rather than inadequate predictor standardization procedures in the included studies.

The Participants domain (D1) showed the only notable proportion of low‐risk studies (19%), indicating that participant data sources were generally well reported. However, many studies did not adequately describe participant inclusion and exclusion criteria, contributing to the 73% unclear risk rating observed in this domain.

Notably, Item 3.6 (time interval) was rated as unclear across all included studies. This likely reflects a limitation of the original PROBAST tool [23], which was primarily developed for traditional prediction models and does not provide a “not applicable” option for cross‐sectional classification studies, such as those investigating ASD. In these studies, predictors and outcomes are typically assessed simultaneously, rendering this item methodologically less applicable rather than genuinely unclear. Recent developments in PROBAST + AI [54] explicitly allow the use of a “not applicable” category for items that may not apply to specific AI/ML‐based prediction models or study designs, thereby improving the flexibility and transparency of ROB assessments. Consequently, the predominance of unclear ratings in this domain may partly reflect limitations of the original PROBAST framework rather than inadequate methodological quality or insufficient predictor standardization procedures.

## 8 Discussion

The ROB assessment of data augmentation studies revealed a predominance of high and unclear risk ratings, particularly within the Analysis domain (D4). As shown in Figure S1, the majority of studies were classified as high risk in the overall assessment, with substantial uncertainty related to overfitting (Item 4.8) and performance evaluation (Item 4.7).

These findings are consistent with the methodological limitations identified across the included studies. In particular, the limited use of ablation experiments, the absence of baseline comparisons, and insufficient reporting of validation strategies contributed to uncertainty in the interpretation of reported performance improvements.

Consequently, although data augmentation techniques were frequently associated with improved model performance, the elevated ROB ratings reduce confidence in establishing a direct causal relationship between augmentation strategies and reported performance gains. These observations are consistent with the findings presented in the data augmentation analysis (Tables 3 and 4). However, the high ROB limits confidence in attributing these improvements to the applied methods.

For example, Xu et al.'s study applied multiple augmentation techniques and evaluated model performance using cross‐validation. However, the analysis focused on overall model performance rather than isolating the contribution of individual augmentation components.

Furthermore, the unclear assessment of overfitting risk suggests that many studies did not adequately evaluate model generalizability, particularly in studies with small sample sizes. These limitations highlight the need for more rigorous evaluation frameworks, including systematic ablation analyses and external validation procedures, to ensure reliable and reproducible findings.

The ROB profile of feature selection studies indicated that most studies were classified as having either high or unclear risk, particularly within the Analysis domain (D4). As illustrated in Figure S2, uncertainty was especially prominent in the assessment of overfitting (Item 4.8) and performance evaluation (Item 4.7).

Similar to data augmentation studies, feature selection studies demonstrated several methodological limitations, including limited use of ablation analyses, absence of baseline comparisons, and insufficient reporting of validation procedures. These limitations contributed to uncertainty in the interpretation of reported performance outcomes.

Feature selection techniques may play an important role in improving model accuracy, generalizability, and computational efficiency in ASD prediction, as reflected in the performance outcomes summarized in Table 5. However, the predominance of high and unclear ROB ratings introduces considerable uncertainty regarding whether the reported performance improvements can be directly attributed to the applied feature selection approaches.

For instance, Bahathiq et al.'s study applied feature selection techniques and validated the model using both cross‐validation and an external dataset [43]. However, the independent contribution of the feature selection stage was not separately evaluated, limiting the ability to attribute performance improvements specifically to the selected features.

Additionally, the predominance of unclear ratings for overfitting risk suggests that model generalizability was not consistently assessed across studies, particularly in studies with small sample sizes. These limitations highlight the need for more rigorous evaluation practices, including systematic ablation analyses and external validation procedures, to improve the reliability and reproducibility of reported findings.

## 9 Conclusions

This review highlights the critical role of data augmentation, feature selection, and model architecture in improving the performance of ML and DL approaches for ASD prediction. Given the heterogeneous nature of ASD, the integration of multimodal data, including neuroimaging, EEG, eye‐tracking, facial expressions, and behavioral assessment data, is essential for developing robust predictive models. This review categorized the major data modalities used in ASD research and examined their contributions to ML/DL‐based predictive pipelines. Despite the richness of these modalities, several challenges remain, including limited sample sizes, class imbalance, and insufficient external validation.

Data augmentation techniques, particularly GANs, have demonstrated considerable potential for addressing dataset scarcity and class imbalance. Although geometric transformations and color‐based augmentations are frequently applied to imaging data, many studies lack systematic evaluation through baseline comparisons and ablation analyses. GAN‐based approaches have shown promising improvements in classification performance for EEG and fMRI data; however, the specific contribution of augmentation strategies is often difficult to isolate because of insufficient comparative evaluation. Furthermore, relatively few studies have explored time‐series augmentation methods for sequential data, limiting the broader clinical applicability of these approaches.

Feature selection represents another essential component of effective predictive model design. Filter‐based techniques, such as information gain and chi‐square, provide efficient dimensionality reduction, whereas wrapper and embedded methods, including RFE and Elastic Net, enable feature selection to be integrated within the training process. Despite their potential to improve predictive performance and interpretability, many studies do not adequately justify the number, stability, or biological relevance of the selected features. This limitation reduces transparency, reproducibility, and potential clinical applicability.

This review also examined predictive modeling strategies used in ASD research. Traditional ML algorithms, including SVM and RF, demonstrated relatively reliable performance across several studies. In contrast, DL architectures, particularly CNNs and LSTM networks, frequently achieved higher predictive accuracy. However, these models often remain susceptible to overfitting, reduced interpretability, and limited efficiency when trained on small datasets.

Transfer learning approaches using pre‐trained architectures, such as ResNet and MobileNet, have further improved predictive performance. Nevertheless, concerns regarding interpretability and model generalizability remain insufficiently addressed. Similarly, hybrid architectures combining deep feature extraction with classifiers such as XGBoost demonstrated promising results but introduced additional model complexity and frequently lacked comprehensive validation procedures.

The risk‐of‐bias assessment demonstrated that many reported performance improvements should be interpreted with caution. Across both data augmentation and feature selection studies, high and unclear risk ratings were primarily associated with limitations in overfitting assessment, insufficient validation procedures, lack of baseline comparisons, and limited use of ablation analyses. These limitations reduce confidence in attributing reported performance gains specifically to the applied methodologies and raise concerns regarding model generalizability and reproducibility.

To improve methodological robustness and clinical reliability, future studies should adopt more rigorous evaluation frameworks, including systematic ablation analyses, explicit baseline comparisons, external validation datasets, and clearly defined cross‐validation protocols.

In addition, the standardized reporting of comprehensive performance metrics, including AUC, *F*_1_‐score, precision, recall, and specificity, is essential for improving comparability and reproducibility across ASD prediction studies. Furthermore, limited interpretability and insufficient justification of selected features continue to restrict the clinical transparency and applicability of many ASD prediction frameworks.

## Author Contributions

**Sahar Alkhaibari:** conceptualization, methodology, investigation, data curation, formal analysis, visualization, writing – original draft, writing – review and editing. **Feng Dong:** conceptualization, methodology, supervision, writing – review and editing.

## Funding

The authors have nothing to report.

## Ethics Statement

The authors have nothing to report.

## Consent

The authors have nothing to report.

## Conflicts of Interest

The authors declare no conflicts of interest.

## Supporting information


Supporting File 1

Supporting File 2

Supporting File 3

## Data Availability

This article is a review and does not generate or analyze any primary datasets. The publicly available datasets and code used in this review were indicated by the original authors, and can be accessed via the references cited in this article. All other data and code are available from the respective authors upon reasonable request.
