# Supplementary material for: Machine Learning for Autism Spectrum Disorder Prediction: A Review of Data Augmentation and Feature Selection Techniques
Source: Health Care Sci. 2026 Sep 7;5(5):514–31. doi: 10.1002/hcs2.70098 (PMC13548289; doi:10.1002/hcs2.70098)
Supplement: Supplementary file 1 [file HCS2-5-514-s001.docx]

Key publicly available datasets used across the reviewed studies are listed below:

- **MRI/fMRI/sMRI:**

**Autism Brain Imaging Data Exchange I (ABIDE I):**

<https://fcon_1000.projects.nitrc.org/indi/abide/abide_I.html>

**Autism Brain Imaging Data Exchange II (ABIDE II):**

<https://fcon_1000.projects.nitrc.org/indi/abide/abide_II.html>

- **EEG:**

**National Database for Autism Research (NDAR), Project: Multimodal Developmental Neurogenetics of Females with ASD:**

<https://nda.nih.gov/>

**Autism Biomarkers Consortium for Clinical Trials (ABC-CT):**

<https://nda.nih.gov/>

**EEG Dataset, University of Sheffield ORD** [55]**:**

<https://doi.org/10.15131/shef.data.16840351>

- **Eye-tracking:**

**Eye-Tracking Dataset to Support the Research on Autism Spectrum Disorder** [56]**:**

<https://doi.org/10.6084/m9.figshare.7073087.v1>

**A dataset of eye movements for the children with autism spectrum disorder** [57]**:**

<https://doi.org/10.1145/3304109.3325818>

**Eye-tracking classification code** [32]**:**

<https://github.com/ai4kids/Eye-Tracking>

- **Facial Images:**

**Kaggle ASD dataset: Autistic children dataset from the Kaggle repository:**

**Google Drive:**

<https://drive.google.com/drive/folders/1XQU0pluL0m3TIlXqntano12d68peMb8A>

**GitHub:**

<https://github.com/mm909/Kaggle-Autism>
