# Supplementary material for: Machine Learning for Autism Spectrum Disorder Prediction: A Review of Data Augmentation and Feature Selection Techniques
Source: Health Care Sci. 2026 Sep 7;5(5):514–31. doi: 10.1002/hcs2.70098 (PMC13548289; doi:10.1002/hcs2.70098)
Supplement: Supplementary file 2 [file HCS2-5-514-s002.docx]

Supplementary Table S1: Related works for ASD prediction research.

| **Author** | **Task** | **Data variables** | **Model** | **Performance** | **Data Augmentation** | **Feature Selection** |
| --- | --- | --- | --- | --- | --- | --- |
| (Lylath & Rananavare 2024) | ASD/Non-ASD | fMRI and the learned attribute graph. | A novel artificial neural network (ANN) + convolutional neural network (CNN) | Accuracy = 78.46  Sensitivity = 81.24  Specificity = 87.43  F-score = 83.8 | None | **Correlation coefficient** |
| (Ladani et al. 2024) | ASD/HC | fMRI data, Sex, handedness, eye status, FIQ, and age | Ridge classifier | ACC = 71.24  AUC = 79.01 | None | **Fisher’s score** |
| (Bahathiq et al. 2024) | ASD/TD | sMRI-based, age, gender | Support vector machine (SVM), naive Bayes (NB), decision tree (DT), random forest (RF), extreme gradient boosting (XGB), category boosting (CatBoost), multilayer perceptron (MLP) | **GWO-based hyperparameter tuning and feature selection algorithms + age and gender:**  **(1) ABIDE I + ABIDE II**  Accuracy = 64.28  Sensitivity = 63.28  Specificity = 61.74  **(2) KAU dataset**  Accuracy = 57.58  Sensitivity = 57.58  Specificity = 53.76 | None | **Recursive elimination** |
| (Nogay & Adeli 2023) | ASD/TD | sMRI data | Grid search optimization (GSO) algorithm is applied to the deep convolutional neural networks (DCNN) | Accuracy (%) = 100 | **Geometric transformations** | None |
| (Chola Raja & Kannimuthu 2023) | ASD/TD | The time series is extracted from fMRI. These characteristics capture dynamic functional connectivity (DFC). | Graph neural network  + conditional generative adversarial networks (C-GANs) | Accuracy = 0.74  Sensitivity = 0.79  Specificity = 0.64 | **Geometric transformations & GAN-based data augmentation** | None |
| (Khan et al. 2020) | ASD/HC | resting-state rfMRI imaging data | A large neural network (TNN) + autoencoder-based neural network model (SNN) + Sequential forward feature selection (SFFS)  + five classifiers (SVM, RF, DT, LR and linear discriminant classifier). | **10-fold accuracy comparison on an all-site combined dataset:**  (1) Classifier name: LR Accuracy = 0.82  Sensitivity = 0.83  Specificity = 0.84  (2) Classifier name: LD Accuracy = 0.82  Sensitivity = 0.83  Specificity = 0.88  **5-fold accuracy comparison on each individual site dataset:**  Classifier name: SVM Accuracy = 0.81 | None | **Forward selection** |
| (Liu et al. 2020) | ASD/HC | resting-state rfMRI imaging data | Support vector machine (SVM) and elastic net (E-net) algorithm | **DATACENTER: LEUVEN**  E-NET:  Accuracy = **83.33** | None | **Regularization** |
| (Xu et al. 2024) | ASD/TD | EEG data (time-series maps of brain functional connectivity (TSM-BFC)) | Convolutional neural network (CNN) and long short-term memory (LSTM) | **Resting EEG**  Accuracy = 81.08%  Sensitivity = 86.32%  Precision = 78.89%  F1 score = 82.37% | **Geometric transformations & GAN-based data augmentation** | **Chi-square test** |
| (Menaka et al. 2024) | ASD/TD | EEG data (spectrograms of 5 cepstral coefficient features) | AlexNet architecture | Accuracy (%) = 90 | None | None |
| (Tang et al. 2024) | ASD/TD | EEG signals data | Hybrid graph convolutional network (RestHGCN) | **Single_subject:**  **average accuracy (%) = 87.16/9.40**  **Cross_subject:**  **average accuracy (%) = 85.32/13.71** | None | None |
| (Lalli & Senbagavalli 2024) | ASD/TD | **Dataset 1:**  EEG signals data  **Dataset 2 (**ABC-CT)**:**  EEG and  eye-tracking data | Hybrid graph convolutional network (RestHGCN) | Accuracies of 91.67 % on the EEG Dataset for ASD and 88.1 % on the ABC-CT dataset. | **GAN-based data augmentation** | **Information gain** |
| (Peng et al. 2021) |  | EEG data | SVM + L1 norm regularization | **L1-regularized SVM under positive emotional stimuli**  Accuracy = 93.8%  **Under negative emotional stimuli**  Accuracy = 87.5% | None | **Regularization** |
| (Wei et al. 2024) | ASD/TD | 89 eye-tracking  features + two demographic variables (age and sex). | **Random forest**, support  vector machine, logistic regression, artificial neural network, and extreme gradient boosting | **RF:** The best performance  AUC = 0.850 ± 0.038,  a sensitivity = 83.1 ± 5.9 %  a specificity = 69.4 ± 7.2 %  an accuracy = 76.9 ± 4.2 % | None | None |
| (Alsaidi et al. 2024) | ASD/TD | 59 children’s eye-tracking scan path images. | Convolutional neural networks (CNN) | **(1) Grayscale images:**  Accuracy = 95.59  Sensitivity = 77.60  Specificity = 79.91  F1-Score = 78.73  **(2) Color images:**  Accuracy = 94.69  Sensitivity = 75.70  Specificity = 75.73  F1-Score = 75.71 | None | None |
| (Islam et al. 2024) | ASD/TD | Visual representation extracted from eye-tracking images from 2 datasets. | Involution fused ConvNet | (1) Dataset-1 Accuracy = 99.43  Recall = 99.30  F1 = 99.49  (2) Dataset-2 Accuracy = 96.78  Recall = 96.29  F1 = 96.58  (3) Merged  Accuracy = 96.32  Recall = 97.63  F1 = 97.42 | **Geometric transformations** | None |
| (Thanarajan et al. 2023) | ASD/Non-ASD | Eye tracking images | ETASD-CBODL (U-Net,  Inception, v3, CBO, and LSTM classification) | Accuracy = 99.29  Precision = 98.78  Sensitivity = 99.29  Specificity = 99.29 | None | None |
| (Kim et al. 2023) | ASD/TD  + ASD symptom severity (severe vs mild to moderate symptoms). | Retinal photographs from the same age and sex | Convolutional neural networks (CNN) with the ResNeXt-50 | (1) ASD vs TD (a):  AUROC = 1.00 (1.00–1.00)  Sensitivity = 1.00 (1.00–1.00)  Specificity = 1.00 (1.00–1.00)  Accuracy = 1.00 (1.00–1.00)  (2) ASD vs TD (b):  AUROC = 1.00 (1.00–1.00)  Sensitivity = 1.00 (1.00–1.00)  Specificity = 1.00 (1.00–1.00)  Accuracy = 1.00 (1.00–1.00)  (3) AUROC = 0.74 (0.67–0.80)  Sensitivity = 0.58 (0.49–0.66)  Specificity = 0.74 (0.67–0.82)  Accuracy = 0.66 (0.60–0.73)  (4) AUROC = 0.44 (0.38–0.50)  Sensitivity = 0.52 (0.46–0.59)  Specificity = 0.44 (0.38–0.51)  Accuracy = 0.48 (0.44–0.53) | None | None |
| (Ahmed et al. 2023) | ASD/TD | A raw statistical dataset of the eye-tracking dataset. | LSTM, CNN-LSTM, Bi-LSTM, and GRU | **BiLSTM**  Test accuracy (%) = 96.44 Sensitivity (%) = 93.50 Specificity (%) = 98.17 AUC (%) = 97  F1 score (% ) = 97.20  **GRU**  Test accuracy (%) = 97.49 Sensitivity (%) = 95.89 Specificity (%) = 98.40 AUC (%) = 97  F1 score (% ) = 98.04  **CNN-LSTM**  Test accuracy (%) = 97.94 Sensitivity (%) = 96.44 Specificity (%) = 98.79 AUC (%) = 98  F1 score (% ) = 98.39  **LSTM**  Test Accuracy (%) = 98.33 Sensitivity (%) = 97.25 Specificity (%) = 98.94 AUC (%) = 98  F1 score (% ) = 98.70 | None | **Information gain** |
| (Khan et al. 2024) | Autistic/Non-autistic | Facial (2D RGB color images in JPEG format) | Five pre-trained DL algorithms: Xception, ResNet50, MobileNetV2, VGG16, and DenseNet121. | **DenseNet-121:**  Precision (%) = 96  Recall (%) = 96  F1 score (%) = 96  Accuracy (%) = 96 | **Geometric transformations** | None |
| (Reddy & J 2024) | Autistic/Non-autistic | Facial (2D RGB color images) | Three pre-trained DL algorithms: VGG16, VGG19, and EfficientnetB0. | **EfficientnetB0: with learning rate [0.001]:**  Accuracy = 88.33%  AUC = 95.44% | **Geometric transformations** | None |
| (Darmiyati, Nugroho & Soesanti 2024) | Autistic/Non-autistic | Six specific landmark points from facial images. | XG Boost, Gradient Boosting, Ada Boost, Decision Tree, KNN, logistic regression, random forest | **Decision Tree:**  **Accuracy = 0.8342** | None | None |
| (P. & V. 2024) | ASD/TD | Facial (2D images) | VGG19, Densenet121, and InceptionV3 | **DenseNet121:**  Accuracy = 96.66%  Precision = 96.25  Recall = 94.75  F1 score **=** 95.50 | None | None |
| (Alam et al. 2023) | ASD/NC | Facial images (2D and 3D) | Five pre-trained DL algorithms: ResNet50V2, VGG19, EfficientNetB0, MobileNetV2, and Xception | **(1) 2D:ResNet50V2**  Accuracy = 100%  **(2) 3D: Xception**  Accuracy = 93.7% | **Geometric transformations** | None |
| (Alam et al. 2023) | ASD/Non-ASD | Facial images. | Three pre-trained DL algorithms: MobileNetV2, ResNet50V2, and Xception. | **Augmentation with pre-processing approach:**  **Xception:**  Accuracy = 98.9  Precision = 98.9  Recall = 98.9  F1-Score = 98.9 | **Geometric transformations** | None |
| (Li, Huang & Song 2023) | Autistic/Non-autistic | Facial features images | **Two-phase transfer learning models + multi classifiers:** MobileNetV2 and MobileNetV3-Large | **Integrated classifier:**  AUC = 0.9647  Accuracy = 0.9050  Sensitivity = 0.9233  Specificity = 0.8867 | None | None |
| (Awaji et al. 2023) | Autistic/Non-autistic | Facial features images | **First:** CNN models (VGG16, ResNet101,  MobileNet) models. **Second: CNN models** (VGG16, ResNet101,  MobileNet) **with ML** (XGBoost and RF) algorithms.  **Third: ML** (XGBoost and an RF) based on features of ( VGG-16-ResNet101, ResNet101-MobileNet, and VGG16-MobileNet) models. | **RF, using the features extracted from VGG16-MobileNet:**  AUC = 99.25%  Accuracy = 98.8%  Precision = 98.9%  Sensitivity = 99%  Specificity = 99.1% | **Geometric transformations & color space transformations** | None |
