# Supplementary material for: Machine Learning for Autism Spectrum Disorder Prediction: A Review of Data Augmentation and Feature Selection Techniques
Source: Health Care Sci. 2026 Sep 7;5(5):514–31. doi: 10.1002/hcs2.70098 (PMC13548289; doi:10.1002/hcs2.70098)
Supplement: Supplementary file 3 [file HCS2-5-514-s003.docx]

**Supplementary Figures**

Figure S1. Risk-of-bias profile of data augmentation studies across key PROBAST domains.

Abbreviations: PROBAST, Prediction Model Risk of Bias Assessment Tool; Std., standardisation; ROB, risk of bias; L, low risk of bias; U, unclear risk of bias; H, high risk of bias.

Figure S2. Risk-of-bias profile of feature selection studies across key PROBAST domains.

Abbreviations: PROBAST, Prediction Model Risk of Bias Assessment Tool; Std., standardisation; ROB, risk of bias; L, low risk of bias; U, unclear risk of bias; H, high risk of bias.
